# The Effect of Snail1 Gene Silencing by siRNA in Metastatic Breast Cancer Cell Lines

**Published:** 2017-05

**Authors:** Mansoor ALETAHA, Behzad MANSOORI, Ali MOHAMMADI, Mehdi FAZELI, Behzad BARADARAN

**Affiliations:** 1. Immunology Research Center, Tabriz University of Medical Sciences, Tabriz, Iran; 2. Dept. of Pathobiology, Shiraz University, Shiraz, Iran; 3. Dept. of Pharmacology and Toxicology, School of Veterinary Medicine, Shiraz University, Shiraz, Iran

**Keywords:** Snail1, siRNA, RNA interference, Cell line, Breast cancer

## Abstract

**Background:**

Breast cancer is the most common diagnosed cancer among women in the world. Snail1 plays a role in the development of the invasive phenotypes of cancer, neural cell differentiation, cell division and apoptosis in tumor cells. Traces of snail1 in metastasis of breast cancer to bone are observed. The aim of this study was to investigate the effect of specific snail1 siRNAs on the proliferation, migration, induction of apoptosis and cell cycle arrest of MDA-MB-468 cells.

**Methods:**

In 2015, this experimental study was performed on the MDA-MB-468 cell lines in Immunology Research Center, Tabriz University of Medical Sciences. After the design and construction of siRNA, transfection was performed with transfection reagent. The expression levels of mRNA and protein were measured by qRT-PCR and western blot analysis, respectively. The survival of cells was determined by using MTT assay cells, apoptosis using Tunel assay, Cell migration using scratch assay, Cell cycle analysis by Propidium Iodide (PI) DNA staining method using flow cytometry on the MDA-MB-468.

**Results:**

Transfection with siRNA significantly suppressed the expression of snail1 gene in dose-dependent manner after 48 h (*P*<0.0001). Surprisingly, treatment with snail1 siRNA arrested cell cycle in S phases (*P*<0.0001). Moreover, siRNA transfection had effects on breast adenocarcinoma cells and inhibited the migration (*P*<0.0001), proliferation (*P*<0.0001) and induced apoptosis (*P*<0.0016).

**Conclusion:**

The snail1 can be considered as a potent adjuvant in breast cancer therapy.

## Introduction

Breast, skin, prostate and colorectal cancers are the most diagnosed cancer types (1, 2). Among all types of cancers, lung cancer is the most killing type in the US (3).

Breast cancer, after skin cancer, is commonly diagnosed and the second type of cancer resulting in death among women in America and first fatal type of cancer among European women (4). In 2012, nearly 200000 American female patients were suffering from metastatic breast cancer, from among whom this cancer was fatal to nearly 40000 patients (5, 6). Annually, the number of death resulted from invasive breast cancer reaches to approximately 500000 cases (6).

Snail factor is a copy of Zinc finger family that plays a role in developing of invasive phenotype in cancer nerve cell differentiation, cell division and apoptosis in tumor cells. The role of snail has been proved in metastatic breast cancer to the bone (7). Snail causes inhibition in protein expression corresponsive to epithelial cells as E-cadherins (the most important factor in cellular connections) (8–10). These proteins are involved in metastasis of the cancers (11). Snail1 is expressed during the formation of mesoderm and development of embryo and formation of blade nervous (12). Cell invasion is an essential step in malignity of cancers that results in fatal metastases. Cell invasion is controlled by some coordinated cellular and molecular events that enable tumour cells to go away from the primary tumour. The changes in cellular connections and immigration of the cell through the tumour remind an important developmental stage named as Epithelial-Mesenchymal Transition (EMT). This event is observed in metastatic cancer cells, too. Cancer cells lose their epithelial feature and cellular connections during EMT and change to the form of mesenchymal and give metastasis to the distant regions (13). Snail expression in metastatic cancer cells and its effect with decrease of E-cadherin has been demonstrated in EMT (14).

According to the previous studied about snail1, it can regulate the expression of several genes which interfere in EMT process. These results indicate that snail1 may be an important regulator during the invasion and metastasis of tumour (15–19). Therefore, snail can play a role in epithelial cancers metastasis.

In this study, we determined snail1 mRNA and protein expression in breast cancer cell line (MDA-MB-468) and analyzed the association of snail1 with cell migration, proliferation, cell cycle and apoptosis in breast cancer cell in vitro.

## Materials and Methods

### Reagents

Cell culture products, MTT Kit, and propidium iodide were purchased from Sigma-Aldrich. (St. Louis, Mo, USA), Design and construction of siRNA, transfection reagent, transfection media and primary antibody were purchased from Santacruz biotechnology. (California, USA), SYBR Premix Ex Taq was purchased from Takara BIO. (Otsu, Shiga, Japan), Protease inhibitor cocktail, ECL Kit, PVDF membrane, In Situ Cell Death Detection Kit and Taq DNA polymerase were purchased from Roche Diagnostics (Gmb.H, Germany), MMLV reverse transcriptase was purchased from Thermo scientific (WI, USA), RNX-PLUS, primer, and DEPC were purchased from Cinnagen (Tehran, Iran), dNTP and buffer PCR were purchased from Fermentas (Helsinki, Finland), RNase. A was purchased from Bioneer. (Daedeok-gu, Daejeon, Korea), horseradish peroxidase-conjugated rabbit anti-goat was purchased from Cyto Matin Gene Company (Isfahan, Iran).

### Cell lines and culture conditions

In 2015, this experimental study was performed on the MDA-MB-468 cell lines in Immunology Research Center, Tabriz University of Medical Sciences. MDA-MB-468 breast adenocarcinoma cell lines were purchased from the Pasteur Institute, Tehran, Iran, and was maintained in RPMI-1640 medium supplemented with 10% FBS, 1% antibiotics (100 unit/ml penicillin and 100 μg/ml streptomycin). Cell lines were cultured at 37 °C in a humidified atmosphere containing 5% carbon dioxide. Every day we changed the culture medium and passaged the cells when they reached 80% to 90% confluences.

### siRNA design and synthesis

Snail1 targeting siRNAs were designed by using Santacruz Biotechnology California, USA (http://www.scbt.com/datasheet-38398-snai-1-sirna-h.html). This designed siRNA sequences were blasted against the human genome database to eliminate cross-silence phenomenon with non-target genes. Scrambled siRNA (Santacruz) that does not target any gene was used as the negative control siRNA.

Pooled human snail1 siRNA is a pool of 3 different siRNA duplexes sequences including siRNA duplex A, Sense: GGACUUUGAUGAAGACCAUtt and Antisense: AUGGUCUUCAUCAAAGUCCtt, siRNA duplex B, Sense: CACGAGGUGUGACUAACUAtt and Antisense: UAGUUAGUCACACCUCGUGtt, siRNA duplex C, Sense: GCGAGCUGCAGGACUCUAAtt and Antisense: UUAGAGUCCUGCAGCUCGCtt.

### Transfection of siRNA

We needed to acquire the time and dosage affected by the siRNA. To do it average dosage of 60 was treated in three times of 24, 48 and 72 h to gain the effective time and three dosages of 40, 60 and 80 pmol were evaluated in the gained effective time to acquire the effective dosage.

Cells were transfected with siRNA and transfection reagent according to the manufacturer’s instructions. Transfection of siRNA was done in three doses of 40, 60 and 80 pmol. Briefly, cells were seeded in a 6-well-plate at a density of 1 × 10^6^ cells/well with antibiotics-free medium 40 min before the transfection. Six μl of the siRNA concentration was mixed with 6 μl transfection reagent in 200 μl optimal medium and were incubated at room temperature for 30 min to form a complex. After rinsing cells with PBS, 212 μl transfection mixtures were supplemented to each well with 800 μl optimal medium. Six hours after the transfection, the medium was replaced with fresh 1 ml RPMI-1640 medium containing 20% FBS. Overall, 24, 48 and 72 h after the transfection, cells was collected for RNA and protein isolation.

### Real-time RT-PCR

RNX-PLUS reagent was used to isolate total RNA from cells according to the manufacturer’s protocol. cDNA was synthesised with 1 μl total RNA (5 ng) using 1 μl random hexamer primer, 1 μl MMLV (Moloney Murine Leukemia Virus), 4 μl 5X reaction buffer and 2 μl of 10 mM dNTP. Five hundred nanograms of cDNA were amplified by real-time PCR using SYBR Green-1 dye universal Master Mix on a LightCycler^®^ 96 System (Roche Life Science, Germany) Sequence Detection System. To confirm the PCR specificity, PCR products were exposed to a melting-curve analysis. The PCR conditions were 95 ^°^C for 10 min followed by 45 cycles at 95 ^°^C for 10 sec, 59 ^°^C for 30 sec and 72 ^°^C for 20 sec relative snail1 mRNA expression were calculated with 2^−ΔΔCT^ method, using β–actin as an internal control. The primers used for the study included: snail1, 5′-GGTTCTTCTGCGCTACTGCTG-3′ (forward) and 5′-GTCGTAGGGCTGCTGGAAGG-3′ (reverse), β–actin, 5′-TCCCTGGAGAAGAGCTACG-3′ (forward) and 5′-GTAGTTTCGTGGATGCCACA-3′ (reverse).

### Western blot analysis

The cell lysate was obtained by using an ice-cold extraction reagent which contains 1 ml triton x-100, 3.3 ml NaCl 5 M, 3.3 ml HEPES 1 M, 1 ml EDTA 0.5 M, glycerol 10% and Protease inhibitor cocktail 1%. The lysate was then centrifuged at 13000 rpm for 20 min at 4 ^°^C and Bradford method was used to determine the concentration of protein. Subsequently, 25 μg of protein samples were separated by 12.5% SDS-PAGE and transferred to the PVDF membrane. After which, primary snail1 antibody was added at a dilution of 1:1000 for a whole night at refrigerator temperature followed by the secondary antibody which was horseradish peroxidase-conjugated rabbit anti-goat at a dilution of 1:5000 for 1 h. For the loading control, monoclonal mouse beta-actin primary antibody at a dilution of 1:3000 was used. The western blots were developed on X-ray films (Eastman Kodak, Rochester, NY, USA) and the optical density of the protein bands was determined after scanning the X-ray film. Densitometry was performed by ImageJ software (National Institutes of Health, Bethesda, Maryland, USA) and the signal intensity of each protein band was normalized to the respective β-actin loading control.

### Detection of DNA Strand Breaks by the Terminal Deoxynucleotidyl Transferase Nick End Labelling (TUNEL) Assay

To determine induction of apoptosis after snail1 siRNA on the MDA-MB-468 cells Tunel assay was used. Cells were cultured at a density of 15 × 10^3^ cell/well in 96-well cell culture plates and then transfected with 60 pmol siRNAs. Forty-eight hours later, the cells fixed with a freshly prepared fixation solution (in 4% paraformaldehyde in PBS, in pH: 7.4), for 1 h at 15 to 25 ^°^C, then slides rinsed with PBS. After that slides incubated with blocking solution (3% H2O2 in methanol) for 10 min at 15 to 25 ^°^C, then slides rinsed with PBS and incubated in permeabilization solution (0.1% Triton X-100 in 0.1% sodium citrate, freshly prepared) for 2 min on ice (2 to 8 ^°^C). Then slides rinsed twice with PBS and dry area around sample. After that added 50-μl Tunnel reaction mixture on sample then added lid and incubated for 1 h at 37 ^°^C in a humidified atmosphere in the dark. Then the slides rinsed three times with PBS. Samples analyzed in drop of PBS under microscope light.

### Proliferation assessment by MTT assay

In this study, proliferation of the treatments was assessed by using MTT assay kit. Briefly, the cells were treated with the agents as described above and incubated in humidified CO2 incubator. Following on 100 ml of MTT reagent (0.5 mg/ml in PBS) was added to each well and then the plates were returned to the incubator for 4 h. The water insoluble formazan crystals were formed during incubation period that solubilized by adding 100 ml of the solubilization (DMSO + Sorenson buffer) to each well. After 30 min incubation in above-mentioned conditions, the absorbances of the solubilized formazan dyes were measured using a Sunrise™ System (Tecan Life Sciences, Austria).

### Scratch assay for migration cells

MDA-MB-468 cells were seeded in a 6-well cell culture plates at 6 × 10^5^ cells. After 24 h incubation, cells reached 100% confluences and scrashed the center of the monolayer by scraping the cells with a sterile 100 μl pipette tip. After scratching, the dish was gently washed with PBS to remove the detached cells, and then transfected with 60 pmol siRNAs. A microscopy system was used to take photographs from the scratch area at 0, 24 and 48 h after scratching (Nikon, Tokyo, Japan), and the cell-free scratch area was measured using NIH Image J software. Quantifying the area covered from the initial time to 48 h-points was used to determine the rate of migration. Multiple views of each dish were examined.

### Flow cytometry assay of cell cycle

Cells were cultured at a density of 2×10^5^ cell/well in 6-well cell culture plates and then transfected with 60 pmol siRNAs. Forty-eight hours later, the cells trypsinized and washed with cold PBS. Then 1 ml cold ethanol 75% was added to the cell plate drop to fix them. The cells were incubated at −20 ^°^C overnight. Then centrifuged at 1000 rpm for 5 min, after which, the supernatant was accumulated. The cells were re-suspended in 500 μl cold PBS and 5 μl of RNaseA was added to each tube and incubate 30 min in 37 ^°^C. Then staining was performed with 20 μl/ml propidium iodide (PI) and 0.1 % Triton X-100. Analysis was done on the part, using FLOWJO software.

### Statistical analysis

Data were expressed in mean value ± S.D. Analysis of variances (ANOVA) or a *t*-test used to analyze the differences between groups. *P-*value less than 0.05 was statistically significant. Using GraphPad prism 6.01 software statistical analyses was performed.

## Results

**Fig. 1:**
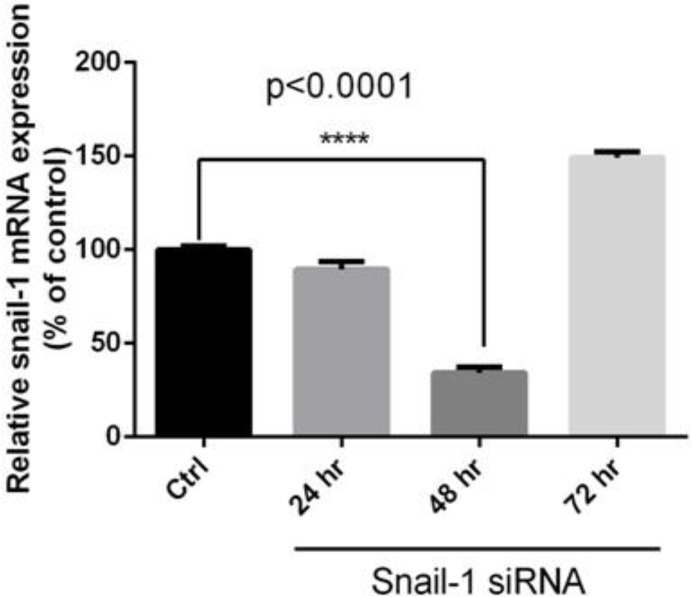
Effective time resulted from the knockdown of snail1 by siRNA in MDA-MB-468 cells Cells were transfected with 60 pM of siRNA as described in methods. At 24, 48 and 72 h post transfection total RNA was extracted and mRNA levels were examined by qRT-PCR. Relative mRNA expression levels were quantified by the qRT-PCR method, using β-actin as an internal control. The data is represented by the mean ± SD (n=3); **** *P*<0.0001 versus control.

**Fig. 2:**
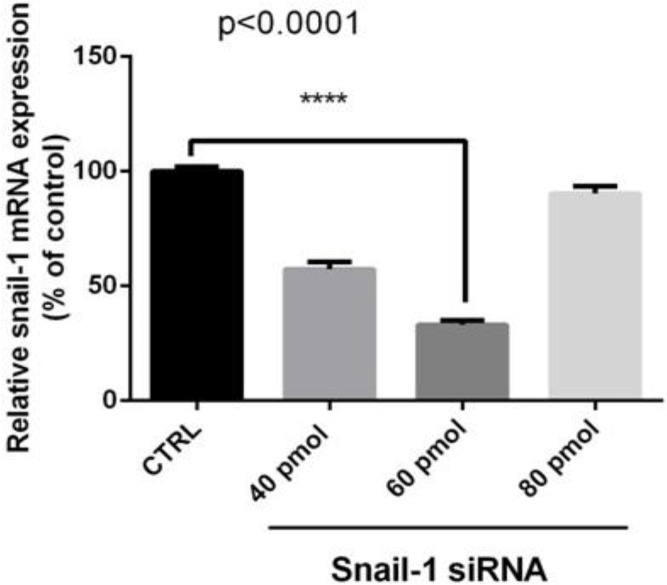
Effective dosage resulted from the knockdown of snail1 by siRNA in MDA-MB-468 cells/Cells were transfected with 40, 60 and 80 pM of siRNA as described in methods. At 48 h post transfection total RNA was extracted and mRNA levels were examined by qRT-PCR. Relative mRNA expression levels were quantified by the qRT-PCR method, using β-actin as an internal control. The data is represented by the mean ± SD (n=3); **** *P*<0.0001 versus control.

Specific siRNAs downregulate snail1 mRNA expression in breast adenocarcinoma cells. We examined the effect of siRNA on mRNA expression in tumor cells. The cells were transfected with siRNA and relative gene expressions were determined using qRT-PCR. The quantitative data gained from each sample was normalized against β-actin and relative gene expression estimated in relation to control (untreated cells), regarding as 100%. Compared with the control, treatment with specific siRNA markedly reduced snail1 mRNA levels in time dependent and dose-dependent manner (*P*<0.05; Fig. 1, 2). At 24, 48 and 72 h post transfection, the relative expression snail1 were 91.33%, 35%, and 152%, respectively, while relative expressions of different concentrations of snail1 siRNA on snail1 mRNA were 58%, 33% and 91%, respectively (*P*<0.05).

The expression levels of β-actin mRNA (served as an internal control for the qRT-PCR) were also similar in all groups (*P*>0.05%). Notably, treatment with NC siRNA (negative control) compared with the control has minimum effect on mRNA levels (*P*>0.05). Specific siRNAs of snail1 could effectively suppress snail1 mRNA expression in breast adenocarcinoma cells, without any specific effects on the β-actin mRNA expression.

### siRNA suppressed snail1 proteins expression in breast adenocarcinoma

**Fig. 3:**
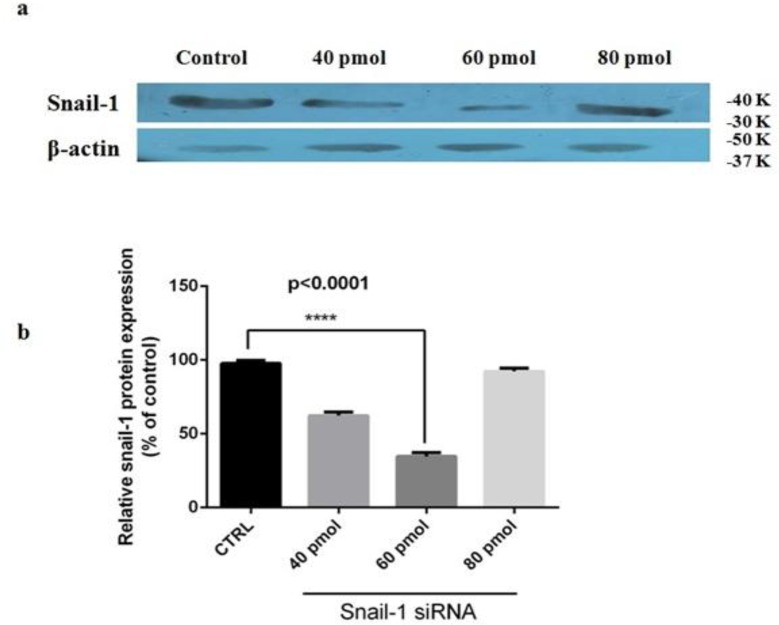
The expression levels of snail1 protein in MDA-MB-468 cells transfected with siRNA, (**a**) Representative western blot of snail1 and β-actin proteins from cells transfected with siRNAs. (**b**) The expression level of each band measured by densitometry and normalized to corresponding β-actin. Results are expressed in relation to the control. The data is represented by the mean ± SD (n=3); **** *P*<0.0001 versus control.

The effects of siRNA on proteins expression were also explored by western blot analysis. Density of each protein band was normalized to the corresponding β-actin and relative gene expression calculated in relation to the control, considering as 100%. As shown in Fig. 3, specific siRNA led to marked dose-dependent reduction of snail1 protein levels (*P*<0.05; relative to control). After the transfection, they were 62%, 35% and 93%, respectively (Fig. 3b; *P*<0.05). The levels of β-actin mRNA and protein were also similar in all groups (Fig. 3a).

### Snail1 suppression decreases cell proliferation in MDA-MB-468 cells in a dose-dependent manner

**Fig. 4:**
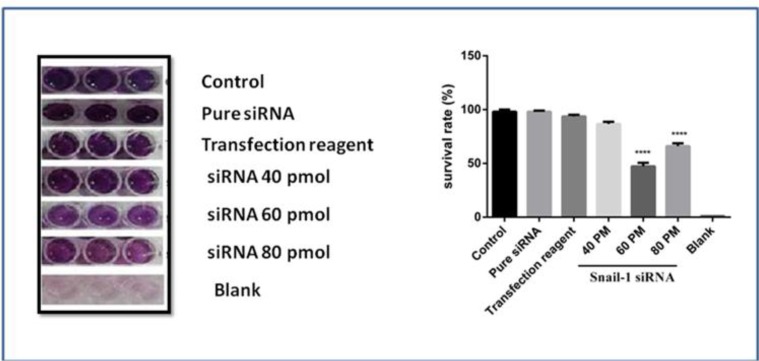
Effect of snail1 siRNA on cell proliferation in MDA-MB-468 cell line. At 48 h after transfection with snail1 siRNA (40, 60 and 80 pmol), as mentioned in methods section survival of treatments was determined by MTT assay. The data is represented by the mean ± SD (n=3). **** *P*<0.0001 versus control

The effect of snail1 downregulation on MDA-MB-468 cells was also investigated. As shown in Fig. 4, mono treatment with specific siRNA of snail1 decreased proliferation in a dose-dependent manner. The results of MTT assay showed that compared with control group, 60 and 80 pmol snail1 siRNA group significantly decreased the cell survival rate (*P*<0.0001).

### Knockdown of snail1 induced apoptosis in breast adenocarcinoma cells

Hallmark of apoptosis is represented by DNA fragmentation. An established method for recognition of DNA fragments is terminal deoxynucleotidyl transferase dUTP end labeling (TUNEL). TUNEL assays detect apoptotic cells by the terminal deoxynucleotidyl transferase (TdT)-mediated addition of labeled (X) deoxyuridine triphosphate nucleotides (X-dUTPs) to the 3′-OH end of DNA strand breaks finally visualized depending on the introduced label, thus it serves as a factor for the percentage of apoptotic cells within the analyzed cell population.

**Fig. 5:**
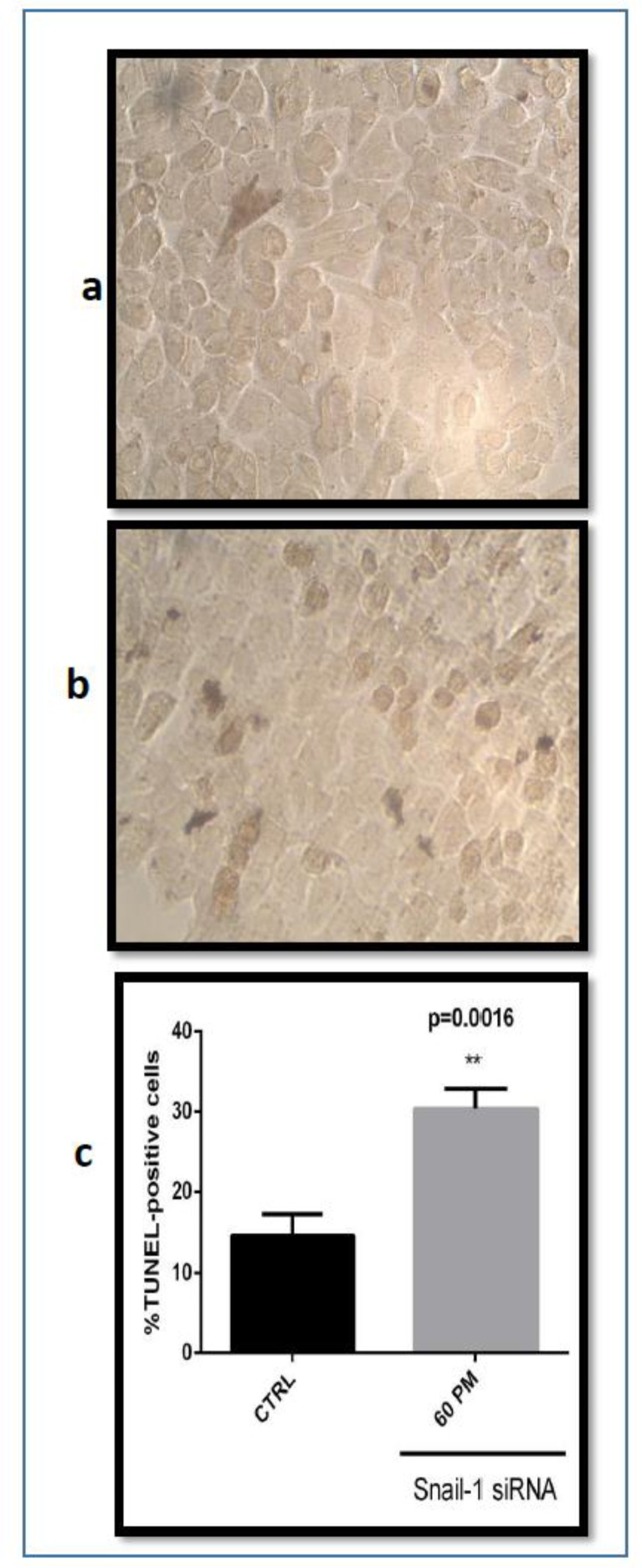
Effect of snail1 siRNA on apoptosis in MDA-MB-468 cell line. (**a**) Untreated siRNA (Control) with the In Situ CELL Death Detection Kit, POD. (**b**) Treated snail1 siRNA with the In Situ CELL Death Detection Kit, POD. (**c**) Percentage of Tunel-positive cells. The results are expressed as mean ± SD (n= 3); ** *P*=0.0016 versus control.

To analyze observed sensitizing effect of snail1 siRNA was linked to the enhancement of apoptosis. When the snail1 siRNA was transfected into MDA-MB-468 cells, the number of surviving cells were decreased at 48 h after transfection and at the dose of 60 pmol snail1 siRNA compared with the control group. The amount of this decrease was impressive (Fig. 5c). The depression in tumor cell number was associated with the appearance of TUNEL positive cells. The nucleus of cells turned brown is apoptotic cells (Fig. 5a, b). The snail1 gene knockdown can induce apoptosis.

### Scratch assay for migration cell

Scratch test for the measurement of cell migration is an easy and low-cost method to study cell-cell interactions in cell migration applied in vitro. Scratch test stages include a scratch on a cell layer and take pictures in the early stages and at different times to observe cell migration in the scratch and to compare the images for evaluation of cell migration.

**Fig. 6:**
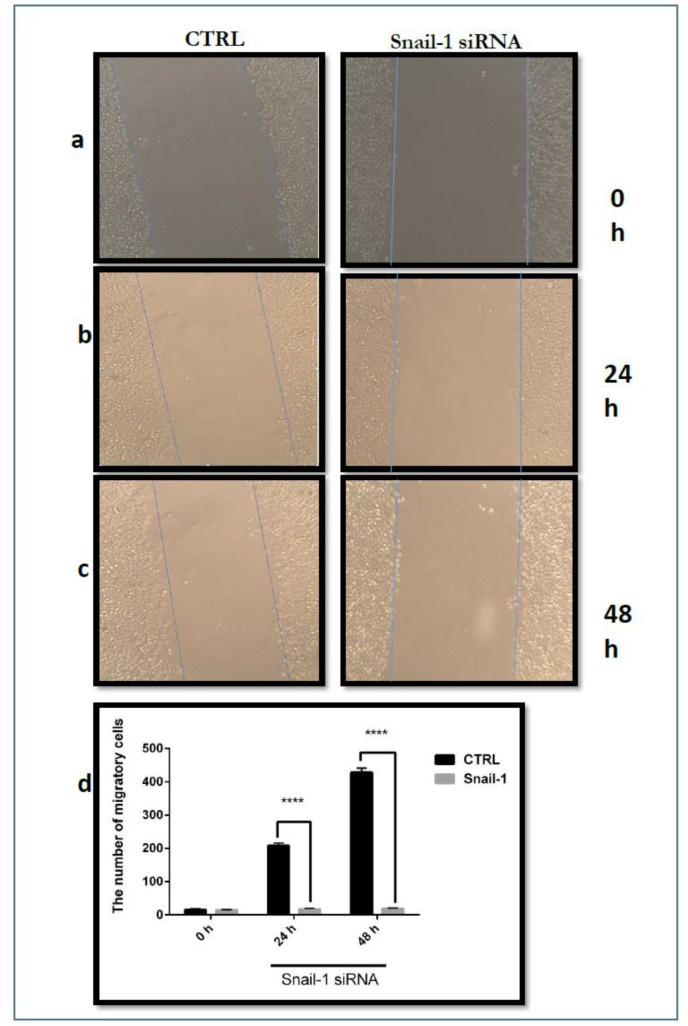
The effect of siRNA snail1 on MDA-MB-468 migration. A confluent monolayer of MDA-MB-468 cells was scratched and then treated with snail1-siRNA. After 24 and 48 h, the scratch area was observed and photographed by microscopy. (**a**) Images at 0 h after transfection of snail1 siRNA and without transfection of snail1 (control). (**b**) Images at 24 h after transfection of snail1 siRNA and control. (**c**) Images at 48 h after transfection of snail1 siRNA and control. (**d**) The quantity was normalized the scratch area between 0 and 48 h against the scratch area at time zero and expressed as number of the migratory rate of MDA-MB-468 cells incubated with the control. Data are presented as means ± SD. (n=3); **** P<0.0001 versus control.

Comparing untreated cells (control) with treated cells by siRNA, snail1 gene silencing led to a significant reduction in breast cancer cell migration (Fig. 6).

### Induction of cell cycle arrest by specific snail1 siRNA

**Fig. 7:**
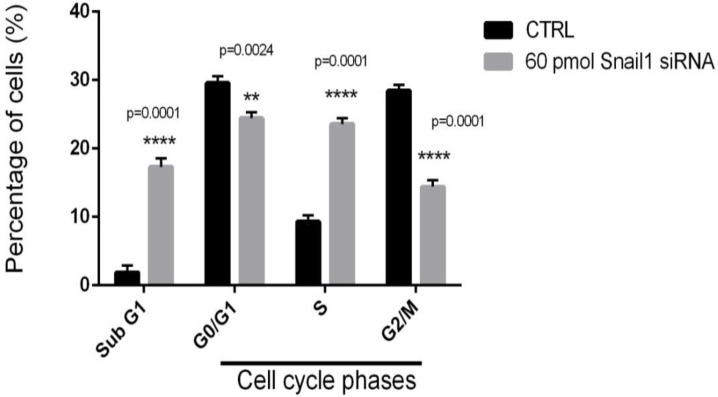
Effect of snail1 knockdown on the cell cycle distribution of MDA-MB-468 cells. Percentages of MDA-MB-468 cells at different phases of the cell cycle following snail1 siRNA or control. **** *P*<0.0001, in comparison to the control.

To analyze observed sensitizing of snail1 siRNA was linked to arresting cell cycle, therefore, we performed fluorescence-activated cell sorting (FACS) analysis. When the snail1 siRNA was transfected into MDA-MB-468 cells, snail1 knock down precluded mitotic entry by inducing G0/G1 arrest and eliminated cells with impaired DNA. We performed FACS analysis to determine the effect of snail1 knockdown on cell cycle distribution. FACS analysis performed 48 h incubation after transfection by 60 pmol specific snail1 siRNA. Cells not transfected by specific snail1 siRNA were used as controls. FACS cell cycle profiles revealed arrest in sub-G1 and S phase in transfected snail1 transfectants during 48 h incubation after transfection of 60 pmol specific snail1 siRNA. At 48 h incubation 17.15% of the MDA-MB-468 cells were in sub G1, 24.26% were in G0/G1, 23.47% were in S and 14.50% were in G2/M while in non-transfected MDA-MB-468 1.97% of cells were in sub G1, 29.93% were in G0/G1, 9.16% were in S and 28.41% were found in G2/M phases (Fig. 7).

## Discussion

The effects of expression and silencing of the snail1 on certain functional properties of MDA-MB-468 cells was demonstrated by the results of the present study. The silencing of snail1 in MDA-MB-468 cell line reduced the snail1 expression level, migration, and cell proliferation significantly and induced apoptosis significantly. In addition, the cell cycle changes of untransfected and transfected MDA-MB-468 cells were assessed and the result showed arrest in subG1 and S phase.

Today, cancer is a human tragedy and deaths from cancer are increased. Cancer results from the cells outgoing of correct regulative paths set for proliferation and differentiation. Malignancy of cancer resulted from growth signals efficacy, insensitivity to growth-inhibitory signals, eluding of programmed cell death, limitless potential of proliferation, angiogenesis maintaining, invasion of tissue and metastasis (20–22).

Breast cancer is one of the most common and fatal cancers among women, just constituting 16% of cancers in women. Per 8 women, one is diagnosed with breast cancer in developed countries (23, 24).

Overexpression of some oncogenes causes tumor progression in many human cancers (25, 26). Some oncogenes not only have the potential to regulate cellular proliferation but also are able to inhibit apoptosis in many types of cancers, including breast cancer (27).

Snail1 has a major role in the development of cancer as a cause of metastasis, inhibition of apoptosis and cell cycle. Snail is a zinc finger transcription factor that plays role in breast carcinomas (28), ovarian (29), melanoma (30), squamous cell carcinoma mouth (31) in the other cancers. In most cancers up-regulation of snail is associated with down-regulation of E-cadherin, which represents the loss of the connection (32). Therefore, the snail is known as an E-cadherin receptor and as a key factor in stimulating EMT. Snail may be associated with the Genesis and development of cancer (33). The expression level of snail was evaluated in stomach, liver, colon, ovarian, and breast cancer cell lines and has been shown that snail inhibits the E-cadherin and induces invasion in phenotype mesenchymal (34–36).

Some studies have been conducted on several types of cancers but there are not many studies about snail1 in breast cancer and its knockdown. Most studies were about snail1 gene expression itself snail1 gene knockdown and not many studies have been conducted about its knockdown and its effect on apoptosis. Snail1 knockdown increases apoptosis that apoptosis induction in cancer cells has a significant importance because apoptosis is one of the ways to fight cancer cells. However, our results are similar to those of others (18, 37, 38).

As we know metastasis in cancer especially in breast cancer has a significant importance because it causes many deaths, so significant decline of the metastases is an important factor. According to previous studies done on prostate cancer cell lines, snail1 knockdown has contributed to multiplication of epithelial forms of cells and decline of invasion and migration of cells (38–40).

Snail overexpression also increased cell migration in MCF7 cells (human breast adenocarcinoma cell line) compared to non-transduced cells (41).

Snail knockdown resulted in significant reduction of the migratory capacity of MDA-MB-468 cells (42). However, our results are similar to these studies.

Snail1 regulates G1 transition (early to late) and the G1/S checkpoint. Flow cytometry analysis indicated that cell cycle arrest in G0/G1 phase resulted from snail overexpression (43).

Mammography is one of the best diagnostic tools for the detection of breast cancer. However, there are restrictions on the use of ionizing radiation and mistakes. The use of conventional markers like estrogen receptor (ER) and growth hormone receptor (HER2) is not flawless (44–46).

Therefore, it is important to identify the suitable markers for early diagnosis of breast cancer for early diagnosis is necessary for effective treatment and on time prevention. However, in the treatment of breast cancer, a gene that plays a role in cancer of the breast can be targeted and by suppression of that oncogene cancer progression can be prevented. Particularly metastasis of cancer can be prevented and deaths from this cancer can be reduced. The results obtained in this study can be commented on snail1 gene that snail1 gene can be either a diagnostic marker or a candidate for treatment of metastatic breast cancer. The final decision on this issue requires additional and vast experiments.

## Ethical considerations

Ethical issues (Including plagiarism, informed consent, misconduct, data fabrication and/or falsification, double publication and/or submission, redundancy, etc.) have been completely observed by the authors.

## References

[1] Sahranavard S Naghibi F Mosaddegh M Esmaeili S Sarkhail P Taghvaei M Ghafari S (2009). Cytotoxic activities of selected medicinal plants from Iran and phytochemical evaluation of the most potent extract. Res Pharm Sci, 4(2):133–7. 21589808 PMC3093631

[2] Valdivieso M Kujawa AM Jones T Baker LH (2012). Cancer survivors in the United States: a review of the literature and a call to action. Int J Med Sci, 9(2):163–73. 22275855 10.7150/ijms.3827 PMC3264952

[3] Winer E Gralow J Diller L (2009). Clinical cancer advances 2008: major research advances in cancer treatment, prevention, and screening—a report from the American Society of Clinical Oncology. J Clin Oncol, 27(5):812–26. 19103723 10.1200/JCO.2008.21.2134 PMC2645086

[4] DeSantis C Ma J Bryan L Jemal A (2014). Breast cancer statistics, 2013. CA Cancer J Clin, 64(1):52–62. 24114568 10.3322/caac.21203

[5] Ahmad Aamir (2013). Breast Cancer Metastasis and Drug Resistance Progress and Prospects. New York.

[6] Izquierdo A Gispert R Saladie F Espinàs JA (2008). Análisis de la incidencia, la supervivencia y la mortalidad según las principales localizaciones tumorales, 1985–2019: cáncer de mama. Med Clin, 131:50–2. 10.1016/s0025-7753(08)76433-9 19080815

[7] Yun J Frankenberger CA Kuo WL (2011). Signalling pathway for RKIP and Let - 7 regulates and predicts metastatic breast cancer. EMBO J, 30(21):4500–14. 21873975 10.1038/emboj.2011.312 PMC3230370

[8] Baritaki S Chapman A Yeung K Spandidos D Palladino M Bonavida B (2009). Inhibition of epithelial to mesenchymal transition in metastatic prostate cancer cells by the novel proteasome inhibitor, NPI-0052: pivotal roles of Snail repression and RKIP induction. Oncogene, 28(40):3573–85. 19633685 10.1038/onc.2009.214

[9] Baritaki S Huerta-Yepez S Sakai T Spandidos DA Bonavida B (2007). Chemotherapeutic drugs sensitize cancer cells to TRAIL-mediated apoptosis: up-regulation of DR5 and inhibition of Yin Yang 1. Mol Cancer Ther, 6(4):1387–99. 17431117 10.1158/1535-7163.MCT-06-0521

[10] Jordà M Vinyals A Marazuela A (2007). Id-1 is induced in MDCK epithelial cells by activated Erk/MAPK pathway in response to expression of the Snail and E47 transcription factors. Exp Cell Res, 313(11):2389–403. 17490644 10.1016/j.yexcr.2007.04.001

[11] De Craene B Gilbert B Stove C Bruyneel E Van Roy F Berx G (2005). The transcription factor snail induces tumor cell invasion through modulation of the epithelial cell differentiation program. Cancer Res, 65(14):6237–44. 16024625 10.1158/0008-5472.CAN-04-3545

[12] Peinado H Olmeda D Cano A (2007). Snail, Zeb and bHLH factors in tumour progression: an alliance against the epithelial phenotype? Nature Reviews Cancer, 7(6):415–28. 17508028 10.1038/nrc2131

[13] Thiery JP Acloque H Huang RY Nieto MA (2009). Epithelial-mesenchymal transitions in development and disease. Cell, 139(5):871–90. 19945376 10.1016/j.cell.2009.11.007

[14] Thuault S Tan E-J Peinado H Cano A Heldin C-H Moustakas A (2008). HMGA2 and Smads co-regulate SNAIL1 expression during induction of epithelial-to-mesenchymal transition. J Biol Chem, 283(48):33437–46. 18832382 10.1074/jbc.M802016200 PMC2662269

[15] Yuan H Kajiyama H Ito S (2013). ALX1 induces snail expression to promote epithelial-to-mesenchymal transition and invasion of ovarian cancer cells. Cancer Res, 73(5):1581–90. 23288509 10.1158/0008-5472.CAN-12-2377

[16] Sun M Guo X Qian X (2012). Activation of the ATM-Snail pathway promotes breast cancer metastasis. J Mol Cell Biol, 4(5):304–15. 22923499 10.1093/jmcb/mjs048 PMC3464396

[17] Zhang A Chen G Meng L (2008). Antisense-Snail transfer inhibits tumor metastasis by inducing E-cadherin expression. Anticancer Res, 28(2A):621–8. 18507000

[18] Olmeda D Jorda M Peinado H Fabra A Cano A (2007). Snail silencing effectively suppresses tumour growth and invasiveness. Oncogene, 26(13):1862–74. 17043660 10.1038/sj.onc.1209997

[19] Sugimachi K Tanaka S Kameyama T (2003). Transcriptional repressor snail and progression of human hepatocellular carcinoma. Clin Cancer Res, 9(7):2657–64. 12855644

[20] Kanellopoulou C Monticelli S (2008). A role for microRNAs in the development of the immune system and in the pathogenesis of cancer. Semin Cancer Biol, 18(2):79–88. 18291671 10.1016/j.semcancer.2008.01.002

[21] Giovannetti E Erozenci A Smit J Danesi R Peters GJ (2012). Molecular mechanisms underlying the role of microRNAs (miRNAs) in anticancer drug resistance and implications for clinical practice. Crit Rev Oncol Hematol, 81(2):103–22. 21546262 10.1016/j.critrevonc.2011.03.010

[22] Li M Li J Ding X He M Cheng SY (2010). microRNA and cancer. AAPS J, 12(3):309–17. 20422339 10.1208/s12248-010-9194-0 PMC2895440

[23] Hickey M Peate M Saunders CM Friedlander M (2009). Breast cancer in young women and its impact on reproductive function. Hum Reprod Update, 15(3):323–39. 19174449 10.1093/humupd/dmn064 PMC2667113

[24] Tavakoli J Miar S Majid Zadehzare M Akbari H (2012). Evaluation of effectiveness of herbal medication in cancer care: a review study. Iran J Cancer Prev, 5(3):144–56. 25628834 PMC4294537

[25] Karami H Baradaran B Esfahani A Sakhinia M Sakhinia E (2014). Therapeutic Effects of Myeloid Cell Leukemia-1 siRNA on Human Acute Myeloid Leukemia Cells. Adv Pharm Bull, 4(3):243–8. 24754007 10.5681/apb.2014.035 PMC3992959

[26] Zaffaroni N Daidone MG (2002). Survivin expression and resistance to anticancer treatments: perspectives for new therapeutic interventions. Drug Resist Updat, 5(2):65–72. 12135582 10.1016/s1368-7646(02)00049-3

[27] Gritsko T Williams A Turkson J (2006). Persistent activation of stat3 signaling induces survivin gene expression and confers resistance to apoptosis in human breast cancer cells. Clin Cancer Res, 12(1):11–9. 16397018 10.1158/1078-0432.CCR-04-1752

[28] Blanco MJ Moreno-Bueno G Sarrio D Locascio A Cano A Palacios J Nieto MA (2002). Correlation of Snail expression with histological grade and lymph node status in breast carcinomas. Oncogene, 21(20):3241–6. 12082640 10.1038/sj.onc.1205416

[29] Imai T Horiuchi A Wang C Oka K Ohira S Nikaido T Ikuo Konishi (2003). Hypoxia attenuates the expression of E-cadherin via up-regulation of SNAIL in ovarian carcinoma cells. Am J Pathol, 163(4):1437–47. 14507651 10.1016/S0002-9440(10)63501-8 PMC1868286

[30] Poser I Domínguez D de Herreros AG Varnai A Buettner R Bosserhoff AK (2001). Loss of E-cadherin expression in melanoma cells involves up-regulation of the transcriptional repressor Snail. J Biol Chem, 276(27):24661–6. 11323412 10.1074/jbc.M011224200

[31] Takkunen M Grenman R Hukkanen M Korhonen M De Herreros AG Virtanen I (2006). Snail-dependent and-independent epithelial-mesenchymal transition in oral squamous carcinoma cells. J Histochem Cytochem, 54(11):1263–75. 16899764 10.1369/jhc.6A6958.2006

[32] Becker K-F Rosivatz E Blechschmidt K Kremmer E Sarbia M Höfler H (2007). Analysis of the E-cadherin repressor Snail in primary human cancers. Cells Tissues Organs, 185(1–3):204–12. 17587826 10.1159/000101321

[33] Espineda CE Chang JH Twiss J Rajasekaran SA Rajasekaran AK (2004). Repression of Na, K-ATPase β1-subunit by the transcription factor snail in carcinoma. Mol Biol Cell, 15(3):1364–73. 14699059 10.1091/mbc.E03-09-0646 PMC363145

[34] Barrallo-Gimeno A Nieto MA (2005). The Snail genes as inducers of cell movement and survival: implications in development and cancer. Development, 132(14):3151–61. 15983400 10.1242/dev.01907

[35] Peinado H Portillo F Cano A (2004). Transcriptional regulation of cadherins during development and carcinogenesis. Int J Dev Biol, 48(5–6):365–75. 15349812 10.1387/ijdb.041794hp

[36] Thiery JP Sleeman JP (2006). Complex networks orchestrate epithelial–mesenchymal transitions. Nat Rev Mol Cell Biol, 7(2):131–42. 16493418 10.1038/nrm1835

[37] Wan Z Pan H Liu S Zhu J Qi W Fu K Zhao T Liang J (2015). Downregulation of SNAIL sensitizes hepatocellular carcinoma cells to TRAIL-induced apoptosis by regulating the NF-κB pathway. Oncol Rep, 33(3):1560–6. 25607597 10.3892/or.2015.3743

[38] Osorio LA Farfán NM Castellón EA Contreras HR (2016). SNAIL transcription factor increases the motility and invasive capacity of prostate cancer cells. Mol Med Rep, 13(1):778–86. 26648419 10.3892/mmr.2015.4585 PMC4686115

[39] Neal CL Mckeithen D Odero-Marah VA (2011). Snail negatively regulates cell adhesion to extracellular matrix and integrin expression via the MAPK pathway in prostate cancer cells. Cell Adh Migr, 5(3):249–57. 21478672 10.4161/cam.5.3.15618 PMC3210209

[40] Jin H Yu Y Zhang T (2010). Snail is critical for tumor growth and metastasis of ovarian carcinoma. Int J Cancer, 126(9):2102–11. 19795442 10.1002/ijc.24901

[41] de Souza Palma C Grassi ML Thomé CH (2016). Proteomic analysis of epithelial to mesenchymal transition reveals crosstalk between SNAIL and HDAC1 in breast cancer cells. Mol Cell Proteomics, 15(3):906–17. 26764010 10.1074/mcp.M115.052910 PMC4813709

[42] Lundgren K Nordenskjöld B Landberg G (2009). Hypoxia, Snail and incomplete epithelial–mesenchymal transition in breast cancer. Br J Cancer, 101(10):1769–81. 19844232 10.1038/sj.bjc.6605369 PMC2778529

[43] Vega S Morales AV Ocaña OH Valdés F Fabregat I Nieto MA (2004). Snail blocks the cell cycle and confers resistance to cell death. Genes Dev, 18(10):1131–43. 15155580 10.1101/gad.294104 PMC415638

[44] Andorfer CA Necela BM Thompson EA Perez EA (2011). MicroRNA signatures: clinical biomarkers for the diagnosis and treatment of breast cancer. Trends Mol Med, 17(6):313–9. 21376668 10.1016/j.molmed.2011.01.006

[45] Heneghan H Miller N Lowery A Sweeney K Kerin M (2009). MicroRNAs as novel biomarkers for breast cancer. J Oncol, 950201. 19639033 10.1155/2010/950201 PMC2712985

[46] Fu SW Chen L Man Y (2011). miRNA biomarkers in breast cancer detection and management. J Cancer, 2:116–22. 21479130 10.7150/jca.2.116 PMC3072617

